# The effect of knowledge on uptake of breast cancer prevention modalities among women in Kyadondo County, Uganda

**DOI:** 10.1186/s12889-018-5183-5

**Published:** 2018-02-23

**Authors:** Christine Atuhairwe, Dinah Amongin, Elly Agaba, Steven Mugarura, Ivan M. Taremwa

**Affiliations:** 1 Institute of Allied Health Sciences, International Health Sciences University, P.O Box 7782, Kampala, Uganda; 2 Mbarara University of Science and Technology, P.O Box 1410, Mbarara, Uganda

**Keywords:** Knowledge, Breast cancer, Modalities, Prevention, Uganda

## Abstract

**Background:**

Breast cancer, the third most frequent cancer of women is preventable through knowledge on breast self-examination. Of the 44% of women diagnosed with breast cancer at the Uganda Cancer Institute, only 22% go for check-up in less than three months. This study explored the effect of breast cancer knowledge on the uptake of breast cancer prevention modalities among women in Kyadondo County, Uganda.

**Methods:**

A household survey of women in Kyadondo County was conducted during June, 2014 to August, 2015. This involved studying in-depth using a questionnaire the level of breast cancer knowledge of the respondents. Data was analyzed using logistic regression model. Chi-square test was used to establish relationships between knowledge base factors and the uptake of breast cancer prevention modalities.

**Results:**

This study has established an empirical relationship between uptake of breast cancer prevention modalities and source of information especially radio (OR 1.94 95% CI: 1.16–3.24), television (OR 1.82 95%CI: 1.14–2.93), awareness of breast cancer (OR 4.03 95%CI: 1.01–15.98), knowledge on how to reduce risk of breast cancer (OR 1.98 95% CI: 1.20–3.27), what reduces breast cancer acquisition (OR 2.75 95% CI: 1.42–5.35), how to check for signs of breast cancer especially through breast self-examination (OR 3.09 95% CI: 1.62–5.88), and other methods of breast cancer diagnosis in a health care set up.

**Conclusion:**

The women’s level of breast cancer awareness as a primary prevention strategy was found wanting, and requires a boost through community health education.

## Background

Breast cancer is the third commonest cancer after cervical cancer and Kaposi sarcoma [1–4]. Worldwide, breast cancer remains the most frequently diagnosed cancer and the leading cause of cancer death among females accounting for 23% of the total cancer cases and 14% of the cancer deaths with a 3% annual incidence and 1.8% death rate [3, 4]. In Uganda is estimated at 4.5% annually as per the age standardized incidence rate but is curable if promptly diagnosed through breast self-examination (BSE) and clinical diagnosis [1]. Breast cancer accounts for 16% of cancer deaths in adult women, and is regarded as a major life threat requiring prompt intervention [2]. Although multifactorial, the high burden of breast cancer is ascribed to its invasive and aggressive nature, and being associated with late presentation to a health facility when it has reached advance stages. Further, the condition is intricated by the fact that its onset is often mistaken for other infections and mismanaged at that level, making it more complex and delayed interventions [5]. This as a result leads to poor prognosis [4, 6].

Breast cancer is preventable through the uptake of such modalities like nationwide breast cancer awareness program involving clinics in remote areas and a referral system that to improve detection and treatment [5, 7]. This approach has been universally considered and it is hoped to subside related morbidities and deaths [7]. As the burden of breast cancer increases, there is minimal awareness in most parts of Africa, and this consequently hinders the possibility of cure, prevention and possible elimination [8, 9]. As a result, this has seen unacceptably high infection cases and deaths among females [8, 10].

As global concerted efforts to upsurge cancer awareness and prevention, there are growing needs to explore knowledge as a measure of disease intervention and possible prevention. To this, we report on the effect of breast cancer knowledge on the adoption of breast cancer prevention modalities among women in Kyadondo County, Uganda.

## Methods

### Design

A household survey of women in Kyadondo County was conducted during the months of June, 2014 to August, 2015. Kyadondo County is a part of Kampala city with a proportion of the urban, peri-urban and rural set up [11]. Kyadondo County was chosen for the study because it is the area from which the Kampala cancer registry captures comprehensive data.

### Sampling procedures

Cluster sampling method was used. Kyadondo County was stratified into 20 parishes [clusters] as the primary stratification variable. At this stage, each parish was further clustered into villages. Each parish comprises 10–20 villages and in each village 50–100 households and the 20 parishes were used as the large clusters from which 10 villages were selected. At the household level, 40 households from each village and one respondent each household was selected randomly giving 400 respondents. Each household had 1–4 women who were aged 18 years of age and above. To minimize bias, 1 woman aged 18 years and above was selected from each household. The age of 18 years was chosen because it is the age of consent and that the person has insight to develop attitudes based on knowledge that may influence her practices.

Inclusion criteria: Women 18 years and above who were able to understand and communicate were included in the study. Only subjects of a sound mind, who were able to hear and speak English and Luganda (a local language used in the area).

Exclusion criteria: participants unable to understand and communicate in English or Luganda or those who declined to consent.

### Data collection and management

Data was captured using a survey questionnaire that was developed from the existing literature on the topic. This was composed in English and then translated to vernacular (Luganda) after being piloted by research assistants. The questionnaire was pre-tested by research assistants among thirty women in Kisugu, Nakawa division to ensure that the most accurate information was obtained and where necessary changes were made. To ensure internal validity, only completed questionnaires were adopted. The questionnaire was interviewer administered.

Study variables: Dependent variable was breast cancer prevention modalities (adopted SBE, clinic breast examination and ultra sound) and the independent variables were background characteristics, knowledge based factors and lifestyle factors.

Knowledge of breast cancer was assessed based on the questions that sought to assess women awareness of breast cancer, knew someone that had breast cancer, risk factors of breast cancer, what to do in order to reduce the risk of acquiring breast cancer, what to do to discover breast cancer early, how to check their own breasts for lumps, and those able to recognize breast cancer signs like discharge from the breast nipple, swelling of the breast among others. The quantification was done as a proportion of the total sample size.

### Data management

Data was entered and analyzed using SPSS IBM version 20. A completely edited dataset was adopted and double checked for errors and corrections done. We considered breast cancer prevention modalities (adopted SBE, CBE and ultra sound) as the dependent variable and the independent variables were background characteristics, knowledge based factors and lifestyle factors. The data was analyzed at univariate, bivariate and multivariate. Variable at univariate and bivariate with a *p* value of less than 0.05 were considered for multivariate analysis and *p* values less than 0.05 were considered statistically significant. Only extract of the bivariate and multivariate were used for this article.

### Ethical considerations

The study was approved by research ethics committee of International Health Sciences University. All respondents provided written informed consent after receiving detailed description of the study. Eligible participants were consented in privacy and no incentives were given. Anonymity of the respondents was ensured at all stages of data analysis.

## Results

### General characteristics of study population

Only 414 subjects were adopted in the study, (that is 85.5% of those who had consent and had complete questionnaires). The results show that 55.3% were below 30 years, 35.5% were 31–50 years and only 9.2% were above 50 years. The religious affiliation revealed that 25.5% were Muslims, 15.5% were Seventh Day Adventists and atheists, 29.5% were Anglican and Roman Catholic respectively. 74.4% of these women were urban dwellers, 20.8% were in semi urban areas and only 4.8% were in rural areas of Kyadondo County. However, the study findings revealed that, 36.2% compared to 63.8% had adopted breast cancer prevention modalities in Kyadondo Count. Among the background factors that were found to be significantly associated with adoption of breast cancer prevention modalities among women in Kyadondo county included: age (*p*-value = 0.049), religious affiliation (p-value = 0.054), level of education (p-value = 0.000) and place of residence (p-value = 0.001). Others background characteristics of the women in Kyadondo County such as distance to the health facility, type of health facility and source of breast cancer information were not significantly associated with the adoption of breast cancer prevention modalities. Only the results that were found to be significantly associated with the adoption of breast cancer prevention modalities are documented in Table 1.

**Table 1:** Background factors associated with adoption of breast cancer prevention modalities among women in Kyadondo County (*n* = 414)

Breast cancer prevention modalities				
Characteristics	Adopted (*n* = 150)	Did not adopt (*n* = 264)	Total	*p*-value
Age				
< 30 years	94(62.7)	135(51.1)	229	0.049^*^
31–50 years	42(28.0)	105(39.8)	147	
> 50 years	14(9.3)	24(9.1)	38	
Religion				
Anglican	40(26.7)	82(31.1)	122	0.054^*^
Roman catholic	54(36.0)	68(25.8)	122	
Muslim	40(26.7)	66(25.0)	106	
Others(SDA etc)	16(10.7)	48(18.2)	64	
Level of education				
None	10(6.6)	42(15.9)	52	0.000^*^
Primary	36(24.0)	126(47.7)	162	
Secondary	52(34.7)	72(27.3)	124	
Tertiary	52(34.7)	24(.1)	76	
Place or residence				
Urban	120(80.0)	246(93.2)	366	0.024^*^
Semi urban	28(18.7)	8(3.0)	36	
Rural	2(1.3)	10(3.8)	12	

*Indicates a *p*-value with a statistical significance

### Breast cancer knowledge of study participants

The study findings indicate that 91.8% of the women were aware of breast cancer, 44.2% knew someone that had breast cancer, only 30.4% knew what increases the chances of breast cancer, 30.4% of the women knew what to do in order to reduce the risk of acquiring breast cancer, more than half 57.5% knew what to do to discover breast cancer early (that is by going for regular check-ups), 35.2% knew how to check their own breasts for lumps, 32.9% of the women had been examined by a health worker and 2.4% had done an ultra sound or had a mammography done prior to the study. In addition, more than half (56.0%) were able to recognize breast cancer signs like discharge from the breast nipple, swelling of the breast among others. The study revealed that 65.7% of the women knew where to obtain breast cancer treatment services in Kampala.

Table 2 indicates the knowledge based factors associated with the uptake of breast cancer prevention modalities among women in Kyadondo County. These include: awareness of breast cancer (*p*-value = 0.001), knowing someone who had suffered from breast cancer (p-value =0.000), obtaining information through health talk shows on radio/television (TV) programs (*p* = 0.004), obtaining information from the health facility (*p* = 0.018), knowing that as one grew older it increased the chances of breast cancer (*p* = 0.000), having a family member that had breast cancer (*p* = 0.003), being obese (*p* = 0.000), eating lots of fatty foods (*p* = 0.000), not giving birth before 35 years (0.005), knowing what would increase one’s chance of acquiring breast cancer (*p*-value = 0.000), knowing how to prevent breast cancer (p-value = 0.000), breast cancer preventions methods like doing exercises daily to control weight (*p* = 0.009), reducing weight if one is obese (*p* = 0.031), eating lots of fruits (*p* = 0.000), reducing alcohol consumption (p = 0.000) avoiding exposure to radiation (*p* = 0.001), knowing how to check for signs and symptoms of breast cancer (*p*-value = 0.000), knowing what to do (for instance a clinical breast exam) (*p* = 0.006), having a breast ultra sound done (*p* = 0.004), having a mammography done (*p* = 0.004), ability to tell the different signs of cancer (*p*-value = 0.000) which included whole breast swelling (*p* = 0.002), swelling in the arm pit (*p* = 0.000) and having an abnormal discharge from the nipple (*p* = 0.000), and knowing where to seek breast cancer treatment services (*p* = 0.000) and at the health care facility (*p* = 0.002).

**Table 2:** Knowledge based factors associated with the adoption of breast cancer prevention modalities

	Breast cancer prevention modalities			
Variable	Adopted (*n* = 150)	Did not adopt (*n* = 264)	Total	*p*-value
Awareness of breast cancer				
Yes	147(98.0)	233(88.3)	380	0.001^*^
No	3(2.0)	31(11.7)	34	
Heard of breast cancer from				
Someone	59(39.3)	117(44.3)	176	0.324
Radio/TV	90(60.0)	120(45.5)	210	0.004^*^
At a health facility/hospital	52(34.7)	63(23.9)	115	0.018^*^
From an outreach	14(9.3)	19(7.2)	33	0.563
Knowing someone with breast cancer				
Yes	85(56.7)	98(37.1)	183	0.000^*^
No	65(43.3)	166(62.9)	231	
Knowing what can increase chances of breast cancer				
Yes	80(53.3)	67(25.4)	147	0.000^*^
No	70(46.7)	197(74.6)	267	
Which of the following can increase someone’s chances of acquiring breast cancer?				
Growing old	22(14.7)	6(2.3)	28	0.000^*^
Having had someone who suffered from breast cancer	24(16.0)	18(6.8)	42	0.003^*^
Having suffered breast cancer before	10(6.7)	10(3.8)	20	0.189
Being obese	12(8.0)	0(0.0)	12	0.000
Eating a lot of fatty food	28(18.7)	10(3.8)	35	0.000
Not giving birth before 35 years	10(6.7)	4(1.5)	14	0.005^*^
Knowing what to do to reduce chances of breast cancer				
Yes	78(52.0)	48(18.2)	126	0.000^*^
No	72(48.0)	216(81.8)	288	
Reducing chances of developing breast cancer				
Doing physical activity/exercise	22(14.7)	18(6.8)	40	0.009^*^
Reducing weight	14(9.3)	10(3.4)	24	0.031^*^
Eating fruits	18(12.0)	6(2.3)	24	0.000^*^
Reducing alcohol consumption	22(14.7)	10(3.8)	32	0.000^*^
Avoiding exposure to radiation	12(8.0)	4(1.5)	16	0.001^*^
Know what to do to discover breast cancer early				
Yes	140(93.3)	98(37.1)	238	0.000^*^
No	10(6.7)	166(62.9)	176	
How to check for signs of breast cancer early				
Check her/his own breasts	102(68.0)	44(16.7)	146	0.000
Be checked by a health worker	62(41.3)	74(28.0)	136	0.006^*^
Have a breast ultra sound scan was done	8(5.3)	2(0.8)	10	0.004^*^
Have a mammography done	8(5.3)	2(0.8)	10	0.004^*^
Able to tell breast cancer signs				
Yes	120(80.0)	112(42.4)	232	0.000^*^
No	30(20.0)	152(57.6)	182	
What shows that one has breast cancer				
Swelling in the breast	84(56.0)	50(18.9)	134	0.000
Whole breast swelling	34(22.7)	30(11.4)	64	0.002^*^
Swelling in the arm pit	14(9.3)	2(0.8)	16	0.000^*^
Discharge from the breast nipple	20(13.3)	8(3.0)	28	0.000^*^
Breast pain	28(18.7)	38(14.4)	66	0.254
How to check for Breast cancer signs				
By someone checking her/his own breasts	32(21.3)	10(3.8)	42	0.000
By health care worker examining breasts	64(42.7)	88(33.3)	152	0.058
By doing a biopsy	44(29.3)	34(12.9)	78	0.000^*^
Don’t know what to do	38(25.3)	106(40.2)	144	0.002^*^
Knew that Breast Cancer can be treated				
Yes	120(80.0)	152(57.6)	272	0.000^*^
No	30(20.0)	112(42.4)	142	
Where BC treatment can be obtained				
Health facility	118(78.7)	170(64.4)	288	0.002^*^
Herbalist	8(5.3)	16(6.1)	24	0.761
Healing Centre/prayer place	4(2.7)	8(3.0)	12	0.832
Don’t know	2(1.3)	14(5.3)	16	0.044^*^

^*^Statistically significant *p* < .05

### Association between knowledge and uptake of breast cancer prevention practices

In order to determine factors that jointly influenced uptake of breast cancer prevention modalities, a binary logistic regression model was fitted. The dependent variable was regressed with a set of independent variables including: socio-demographic characteristics and knowledge base factors. The dependent variable was dichotomous and grouped as 1-adopted and 2-did not adopt. The results in Table 3 indicate that subjects who had primary level of education were 5 times and those with secondary education were 3 times more likely to adopt breast cancer prevention modalities respectively. The respondents who were aware of breast cancer were 1.5 times more likely to adopt breast cancer prevention modalities than their counterparts. The study findings revealed that the following increased the chances of acquiring breast cancer: ageing (OR 2.2, CI .53–9.59), having someone in the family with a history of cancer (OR 1.20, CI .31–5.07), eating fatty foods (OR 0.25, CI .06–1.09), drinking alcohol (OR 1.1, CI .33–4.08). It was also established that knowing what to do reduced the risk of breast cancer (OR 2.16, CI .92–5.07). When asked to identify what actually reduces risk of acquiring breast cancer, the women in Kyadondo identified the following: eating plenty of fruits (OR 0.10, CI .01–.85), reducing alcoholic consumption (OR 0.19, CI .05–.78) and avoiding exposure to radiation (OR 0.47, CI .05–4.39). The respondents who knew what to do to reduce their chances of acquiring breast cancer were 2.15 times more likely to adopt breast cancer prevention modalities.

**Table 3:** Adoption of breast cancer prevention modalities: A summary of the findings

Characteristics						95% C.I. for EXP(B)	
Background factors	Coefficient B	Standard Error S.E.	Wald	Sig.	OR Exp(B)	Lower	Upper
Age							
< 30 years	.276	.701	.155	.693	1.32	.33	5.21
31–50 years	.297	.665	.200	.655	1.35	.37	4.96
Religion							
Anglican	−.094	.557	.029	.865	.91	.31	2.71
Roman catholic	−.309	.567	.298	.585	.73	.24	2.23
Muslim	−.152	.546	.077	.781	.86	.30	2.50
Education			11.310	.010			
None	1.043	.619	2.837	.092	2.84	.84	9.55
Primary	1.710	.516	10.960	.001	5.53	2.01	15.21
Secondary	1.322	.505	6.862	.009	3.75	1.40	10.09
Residence			8.309	.016			
Urban	−2.095	1.146	3.342	.068	.12	.01	1.16
Semi urban	−.887	1.145	.600	.438	.41	.04	3.88
Knowledge base factors							
Heard of Breast Cancer (*yes)*	.437	.868	.254	.614	1.55	.28	8.49
Told by someone (yes)	−.051	.377	.018	.893	.95	.45	1.99
Health facility (*yes*)	.075	.398	.035	.851	1.08	.49	2.35
Knew someone with BC(*yes*)	−.071	.341	.043	.835	.93	.48	1.82
Risk of breast cancer	−1.080	.393	7.547	.006	.34	.16	.73
Growing old(yes)	.814	.738	1.214	.271	2.26	.53	9.59
Family history of BC(*yes*)	.218	.716	.093	.760	1.24	.31	5.07
Eating fatty foods(*yes*)	−1.389	.750	3.424	.064	.25	.06	1.09
Drinking alcohol (*yes*)	.141	.645	.048	.827	1.15	.33	4.08
Know how to reduce chances of BC(*yes*)	.769	.436	3.115	.078	2.16	.92	5.07
Eating fruits (*yes*)	−2.280	1.080	4.461	.035	.10	.01	.85
Reduce alcohol intake (*yes*)	−1.650	.716	5.308	.021	.19	.05	.78
Avoid radiation (*yes*)	−.748	1.136	.434	.510	.47	.05	4.39
Know how to check for signs of BC(yes)	−3.719	.509	53.428	.000	.02	.01	.07
Clinical breast examination (*yes*)	.913	.355	6.625	.010	2.49	1.24	4.10
Have breast ultra sound (*yes*)	.894	1.671	.286	.593	2.45	.09	64.68
Have a mammogram done (*yes*)	.172	1.173	.021	.884	1.19	.12	11.82
Know all signs of BC(*yes*)	−.486	.475	1.048	.306	.62	.24	1.56
Small swelling on the breast (yes)	−1.039	.430	5.854	.016	.35	.15	.82
Swollen breast (yes)	.328	.542	.366	.545	1.39	.48	4.01
Abnormal nipple discharge (*yes*)	−1.295	.708	3.345	.067	.27	.07	1.10
Self breast examination (*yes*)	−1.423	.671	4.494	.034	.24	.07	.90
Examined by a Clinician (yes)	−.361	.583	.383	.536	.70	.22	2.18
Doing a biopsy (yes)	−.478	.660	.525	.469	.62	.17	2.26
Don’t know what to do	−.320	.625	.261	.609	.73	.21	2.48
Availability of BC treatment (yes)	.653	.487	1.799	.180	1.92	.74	4.99
Provided at the health facility (yes)	−.303	.459	.435	.510	.74	.30	1.82
Constant	4.424	1.686	6.887	.009	83.45		

−2 Log likelihood 294.388

Cox & Snell R^2^ 0.450

Nagelkerke R^2^ 0.617

A woman’s knowledge on the signs of breast cancer was analyzed in relation to adoption to breast cancer prevention modalities. The breast cancer signs identified include: small swelling on the breast (OR 0.35, 95% CI .15–.82), swollen breast (OR 1.39, 95% CI 48–4.01) and abnormal nipple discharge (OR 0.27, 95% CI .07–1.01), However it was established that the women in Kyadondo who were able to identify the signs of breast cancer were 0.61 times less likely to adopt breast cancer prevention modalities than their counterparts. When asked what can be done to discover breast cancer early: the women were able to identify the following: self-breast examination (OR 0.24, 95% CI .07–.90), clinical breast examination (OR 0.70, 95% CI .22–2.18), doing a biopsy (OR 0.62, 95% CI .17–2.26), and those who did not know what to do (OR 0.73, 95% CI .21–2.48). Finally, our respondents indicated that breast cancer treatment facilities were likely to adopt prevention modalities (OR 1.92, 95% CI .74–4.99). The results revealed that women who acknowledged that there was breast cancer treatment at the health facility were 0.73 times less likely to adopt breast cancer prevention modalities than their counterparts.

## Discussion

Breast cancer-preventive modalities are critical for the health of women and their communities. The odds of adopting breast cancer prevention modalities were higher among women who had none formal education, primary education and secondary education than tertiary education in Kyadondo County.

The odds of adopting breast cancer prevention modalities were more likely for a woman who had general knowledge on breast cancer than if she did not. This is similar to what was revealed in a study of women’s knowledge toward breast cancer that established that the respondents who had little or no preventive breast cancer information were less likely to know how to prevent breast cancer [11]. The study results established that chance of a woman adopting breast cancer prevention modalities were less if she heard it from someone in the community. There were moderate chances of a woman in Kyadondo county adopting breast cancer prevention modalities if she went to the health facility to get information on breast cancer.

The odds of a woman adopting breast cancer prevention modalities were less likely if she knew someone who had suffered from breast cancer. This is an indicator of a deficit in the knowledge of breast cancer which is in line with the findings on breast cancer screening practices among Ghanaian women where higher knowledge was linked to better management (rs = 0.316, *N* = 465, *p* < 0.001) [7].

The odds of adopting breast cancer prevention modalities were higher for a woman who was growing older than their younger counterparts. The chances of a woman in Kyadondo county adopting breast cancer prevention modalities were moderate among women with a family history of the disease.

Knight in his study on breast cancer reported that the consumption of alcohol in large quantities increases the risk of developing breast cancer [10]**.** Further, a study conducted by Merriman argues that women who do not have children or who had their first child after age 30 have a slightly higher breast cancer risk of acquiring breast cancer [8]. The findings also revealed that the odds of adopting breast cancer prevention modalities were higher for a woman who knows what reduces the chances of acquiring breast cancer. This means a woman is likely to take precaution against breast cancer if she knew the benefits of exercising, reducing weight, eating fruits, reducing alcoholic consumption and avoiding exposure to radiation. Ahmadian upholds this by advising adults to eat healthy foods, take a glass of wine occasionally, exercising like having a 25 min’ walk every day or have at least 150 min a week of moderate aerobic activity [12].

The study indicates that the odd of adopting breast cancer prevention modalities were higher if a woman had knowledge on the signs of breast cancer than if she did not. The odds of adopting breast cancer prevention modalities were higher for a woman who knew what to do to discover breast cancer early (self-breast examination, clinical examination, breast ultra sound and mammography) than for someone who did not. Merriman observed that women with a better knowledge level on breast cancer (Adjusted Odds 2.5) where more likely to adopt BSE [8].

The results revealed a strong relationship between adopting breast cancer prevention modalities and exercising. This means that the more a woman exercises the lower the chances of her acquiring breast cancer. Lemanne provides evidence of the benefits of exercising that included: improves cardiovascular (heart and circulation) fitness and that exercise helps one gain a healthy weight; made the muscles stronger; reduces fatigue and helps people have more energy; helps lower anxiety and depression; makes one feel happier and be better about oneself as well as long term effects of reducing breast cancer [13].

The odds of adopting breast cancer prevention modalities was more likely if a woman was able to identify signs of cancer like a small swelling on the breast than if she did not. It was established that women who had a strange discharge from the breast may signify cancer were 8 times more likely to adopt breast cancer prevention modalities than if they were not. This concurs with Merriman who argues BSE is a patient-centred, non-invasive screening procedure that allows women to routinely check their bodies [8]. In addition the odds of adopting breast cancer prevention modalities were more likely if a woman was able to examine her breast than if she did not. The women who acknowledged that there is treatment of breast cancer had higher chances of adopting breast cancer prevention modalities than their counterparts. This affirms the findings among female undergraduate students in Buea where a gap in knowledge was established in the practice BSE in the prevention of breast cancer [14]. Therefore this study is in agreement with what Fon already reported that acquiring cancer knowledge, cancer detection and treatment among women increases women’s practice of BSE [7]. This may enable a woman to make an informed decision about breast cancer screening risks and the benefits.

Although our findings are factual, it falls short of the following; a) being a field based survey, our findings solely relied on the respondents have given us accurate information; otherwise the study did not have a confirmatory basis to validate the trueness of their responses. b) this study could have been subjective to forms of bias particularly recall bias, in addition, since we used an interviewer-administered questionnaire, it is likely that interviewer bias was encountered, c) since our study targeted a single adult female respond as a proxy to each household, it is likely some selection bias was encountered.

## Conclusion

Breast cancer awareness ought to be rolled out particularly to the school girls with emphasis on how to conduct breast self-examination and where to obtain a clinical breast exam so as to minimize late breast cancer detection and treatment at stage 4. In addition, health programs in form of the media coverage through newspapers, local written and oral, radio and television could alleviate breast cancer awareness. Through these, it is hoped to harness awareness of breast cancer, the need of its early diagnosis, as well as prompt treatment.

## Abbreviations

BCE: Breast clinical examination
BSE: Breast self-examination
OR: Odds Ratio
